# Ethical challenges in surgery as narrated by practicing surgeons

**DOI:** 10.1186/1472-6939-6-2

**Published:** 2005-02-28

**Authors:** Kirsti Torjuul, Ann Nordam, Venke Sørlie

**Affiliations:** 1 Sør-Trøndelag University College, Faculty of Nursing, Trondheim, Norway; 2 Centre for Medical Ethics, University of Oslo, Norway; 3 Institute of Nursing and Health Sciences, Faculty of Medicine, University of Oslo, Norway

## Abstract

**Background:**

The aim of this study was to explore the ethical challenges in surgery from the surgeons' point of view and their experience of being in ethically difficult situations.

**Methods:**

Five male and five female surgeons at a university hospital in Norway were interviewed as part of a comprehensive investigation into the narratives of nurses and physicians about being in such situations. The transcribed interview texts were subjected to a phenomenological-hermeneutic interpretation.

**Results:**

No differences in ethical reasoning between male and female surgeons were found. They reasoned in both action and relational ethical perspectives. Surgeons focused on their relationships with patients and colleagues and their moral self in descriptions of the ethical challenges in their work. Dialogue and personal involvement were important in their relationships with patients. The surgeons emphasized the importance of open dialogue, professional recognition, and an inclusive and accepting environment between colleagues.

**Conclusion:**

The surgeons are personally challenged by the existential realities of human life in their relationships with patients. They realized that ethical challenges are an inherent part of performing surgery and of life itself, and say that they have to learn to "live with" these challenges in a way that is confirmed both socially and by their inner moral self. This means accepting their personal and professional limitations, being uncertain, being fallible, and being humble. Living with the ethical challenges of surgery seems to contribute to the surgeons' confidence and vulnerability in their professional identity.

## Background

It is important for surgeons to be and to act in a right and good way towards patients, relatives, and colleagues. Studies have shown, however, that physicians often are in doubt about the best and correct actions to take for the patients in specific situations [1-3]. This question is not only a medical one, but can be understood in both action ethics and relational ethics perspectives. An action ethics perspective concerns questions as to what ought to be done in ethically difficult situations and why. In this perspective, ethics often centres on difficult ethical dilemmas and decision-making. Ethical dilemmas occur when physicians have to choose between at least two alternative and equally difficult courses of actions. Because neither of the alternatives have positive outcomes, they have to choose between two evils [4]. Ethical dilemmas can also be understood as conflicts between different courses of action that result from general and mutually exclusive ethical principles in medicine [5].

A relational ethical perspective means reflecting on the challenges we encounter in our relationships with others and how to fulfil our social roles and obligations in a good way – as a human being, a surgeon, and a colleague. It tries to answer questions such as "How can I adequately meet the challenges that confront me in the relationships in which I am involved in this situation?" [4]. The qualities that make a person a good physician are not only individual traits but they are characteristics of the relationships. One way to describe a good physician or a surgeon is to count the number of characteristics or virtues which are portrayed. According to MacIntyre [6], another way is to speak about a good physician or a surgeon. Narrative ethics focus on what life demands from us in different situations and how we ought to respond to these challenges [4,6,7].

Action and relational ethical perspectives are not interchangeable as surgeons have a dual responsibility for their actions in specific situations as well as their way of being in their relationships [4,7]. Being a good surgeon presupposes both professional competencies based on scientific and clinical knowledge and skills, and being present and showing respect and compassion for patients [4,5,8]. Physicians become involved in the patients' problems both in a professional and moral sense [9]. Traditionally, it has been assumed that compassion can impair competence and that they cannot coexist [10].

Many authors argue that the respect and trust in the physician-patient relationship have eroded in recent years in spite of the physicians' increased therapeutic capabilities [11,12]. Shorter hospital stays and organizational changes in the hospitals are said to lead to surgeons spending more time in the operating theatre and less time talking to patients and establishing a trusting relationship [7,13]. An open and honest dialogue between physicians and patients can be difficult to achieve as medicine becomes more complex, fragmented, episodic, and impersonal, according to Jones [14].

Medical problems are always existential problems too because suffering, anxiety, life, death, and cure involve the core of human existence. Physicians are working with emotionally intense issues and have to accept the possibility of failures, continuing suffering, and death on the part of their patients [12]. Meeting patients who are emotionally distressed or tragically injured can make surgery emotionally challenging [15]. Patients may also elicit emotions of anger and frustration, fear, and despair in physicians [16]. Research suggests that the delivery of bad news can be particularly troubling for both patients and physicians because of the emotional component, and that physicians experience great discomfort in such situations [17]. Physicians have to function at an optimal level despite these challenges. Straume [18] regards the physicians' vulnerability as a result of their overwhelming responsibility and experience that patients' demands often exceed the physicians' ability.

Although most clinicians are aware of the uncertainty and the limitations of medicine and their responsibility to try to reduce the likelihood of error, [19,20] the boundary between medical errors and accidents is not evident [21-23]. Physicians may have difficulty acknowledging personal errors because they can be experienced as personal defeats and thus confirm that physicians are vulnerable [20,24].

Surgeons' relationships with their colleagues have become more important as surgery has changed from relying heavily on the performance of individual surgeons to relying on a team of providers [8,25]. Several studies have found a lack of dialogue and support structures among physicians [26,27]. Physicians are said to have no tradition for open discussions about uncertainty and conflict areas in their practice, nor are they comfortable talking openly about their personal emotions and problems [12,16,24]. Adverse events are said to be generally managed by the conspiracy of silence [20,22].

The vulnerability of patients is emphasized in the literature of medical ethics. Less is written about the vulnerability of the physicians in their relationships with patients, relatives, and colleagues. Being involved may engender feelings of helplessness and vulnerability in the physician. MacLeod [28] argues that physicians have to accept their vulnerability and be able to express and share it in order to be able to live with the tensions. Little [29] suggests that understanding the peculiarities and intensity of the patient-surgeon relationship may help the surgeons understand the vulnerability of both patients and surgeons.

Studies show that physicians seem to experience uncertainty and fallibility in different ways. Less experienced female physicians in paediatric care put on an air of certainty while the more experienced gained a kind of security by allowing themselves to feel uncertain [30]. Female physicians in geriatric care seemed to accept their own vulnerability and fallibility [31]. Experienced male physicians in pediatric care related personal security to their professional experience while the less experienced thought that advances in medical knowledge and ethical guidelines would make them more secure in their work [32]. Henriksen and Hansen [33] found that general practitioners seemed to strive for the ideal of a humble attitude towards problems; being too self-confident was regarded as a threat because of the increased risk of downfall.

Few empirical studies have been found that explore the ethical challenges of surgery from the surgeons' point of view and their experience of being in ethically difficult situations. The present study is part of a comprehensive investigation of ethical reasoning among male and female physicians and nurses within surgical units. The results of this interview study with male and female surgeons will be presented in two articles. The present study describes the surgeons' experiences of being in ethically difficult situations from a relational ethics perspective. The other paper describes the ethical dilemmas as experienced by the surgeons from an action ethical perspective. The results from the interviews with the registered nurses (RNs) working in surgical units are in progress and will be addressed in a third paper.

The aim of this study is to explore the meaning of being in ethically difficult situations in surgery as narrated by male and female surgeons.

## Methods

### Participants and setting

Five male and five female surgeons working on surgical units at a university hospital in Norway participated in the study. All were experienced and had been working in health care from 9 to 31 years (median = 21.5), and in surgery between 5 to 21 years (median = 13). The surgeons worked full time and were on duty when the interviews were conducted. No individual characteristics will be disclosed in order to guarantee confidentiality. The surgeons gave their informed consent to participate in the study, which was also approved by the 5^th ^Regional Ethics Committee in Norway.

### Data collection

#### Interviews

The interviews were conducted by the first author and lasted from 35 to 75 minutes (median = 55). They were tape recorded and subsequently transcribed verbatim. The interviewees were asked to tell about one or more ethically difficult care situations that they had experienced in their work as surgeons. What constituted an ethically difficult situation was not defined, allowing the interviewees to determine what they considered ethically difficult themselves. The aim of the interviews was to obtain as many rich narratives as possible without interrupting the surgeons' narrative flow and reflection. If the surgeons did not spontaneously reflect on the events they talked about, their reflections were sought. Questions were asked when the interviewer wanted the interviewees to elaborate on their stories or had difficulty understanding the narration. These questions referred to the interviewees' thoughts, feelings, and actions [34]. Field notes were taken during the interview as aids to the interviewer's memory and in order to make it possible to understand the interview text in relation to its context, e.g. arrangements and interruptions. Nonverbal communications that seemed relevant were also noted, such as laughter and long pauses. The transcribed text was compared with the field notes and adjusted if necessary.

### Data analysis

#### Interpretations

The method of interpretation used was inspired by the French philosopher Paul Ricoeur's phenomenological hermeneutics [35], and developed at the University of Tromsø (Norway) and Umeå University (Sweden) and has previously been used by Lindseth et al., [36] Udén et al., [1,13] Søderberg et al., [37] and Sørlie et al. [30,32,38]. This method is useful to elucidate the narratives of people's experiences. The method of interpretation proceeds through three phases, which constitute a dialectical movement between the whole and the parts of the text and between understanding and explanation [35].

Each interview was regarded as a text. However, it was not what the texts said that was a subject matter to be investigated, but rather the focus was on the ethics expressed in them or the essential meaning of ethically good phenomena (or the essential meaning missing in ethically poor phenomena)[39]. First, a *naïve reading *was made of all the transcribed interviews as a whole to gain a first impression of the surgeons' experiences of being in ethically difficult situations during their clinical work. The repeated naïve reading was made as open-minded as possible, without any deliberate analysis of the text. The naïve reading shows the direction the structural analysis may take. Second, a *structural analysis *was performed in order to validate or refute the initial understanding obtained from the naïve reading and to explain what the text was saying. The interviews were divided into meaningful parts and patterns, i.e. one sentence, parts of a sentence, or a whole paragraph with a related meaning content. The meaning units were condensed and discussed among all the authors, and themes and subthemes were identified, and presented in 'Results'. Third, a *comprehensive understanding *was developed, taking into account the authors' pre-understanding, the naïve reading, and the structural analysis (results). The text was read as a whole and interpreted in relation to relevant theories of ethics and results from previous investigations into the meaning of being in ethically difficult care situations [39]. The comprehensive understanding is presented under the heading 'Discussion'.

The analysis was conducted by all the authors and the interpretative agreement was considered satisfactory and to be the most useful understanding of the meaning of the surgeons' experiences of situations of ethical difficulty. The authors' interpretation was not shared or validated with the surgeons. In this study, the focus was on obtaining the meaning of the text, which cannot be validated by the interviewees. A kind of validation is accomplished by the structural analysis as the objective part of the interpretation process [39]. According to Ricoeur [35] a text has multiple but not infinite meanings. One particular phenomenological hermeneutic interpretation should therefore be seen as one of several possible interpretations and as arguments put into ongoing discourses, in this case, about ethical challenges in surgical care.

## Results

Several readings of the interview texts revealed that the surgeons told about ethical challenges that confront them in their relationships with patients and colleagues and about their experiences of living with these challenges. They also reported their experiences of ethical dilemmas in surgical practice. No gender differences between the male and female surgeons were found in the analysis of the interviews. The results showed that each surgeon created many and detailed narratives. When the surgeons were asked to narrate their experiences, they did not differentiate between action and relational perspectives in their ethical reasoning. This is an analytical distinction made by the authors in order to structure the results. The authors therefore decided to separate the presentation of the results in two papers: one paper about the surgeons' experiences of being in ethically difficult situations from a relational ethics perspective and the other paper from an action ethical perspective according to the theory presented in the introduction [4]. This paper presents the ethical challenges of surgery as narrated by five male and five female surgeons.

The themes and the subthemes from the structural analysis are shown in Table 1 and presented in the text below. Direct quotations from the interviewers are included to illuminate the results.

**Table 1:** Themes and subthemes that emerged from the structural analysis of interviews with the surgeons.

**Themes**	**Subthemes**
**Dialogue with patients**	*Openness and honesty*
	*Involvement*

**Social confirmation**	*Professional recognition*
	*Open dialogue*

**Self confirmation**	*Responsibility*
	*Uncertainty*
	*Fallibility*
	*Confidence*
	*Humility*

### Dialogue with patients

#### Openness and honesty

The surgeons emphasized the importance of dialogue with patients and especially being open and honest about all aspects of their treatment and care. Talking to patients about difficult issues before or after the operation is experienced as an important part of the surgeons' responsibility. Openness and honesty is especially important when surgeons had to tell patients that they have cancer or a fatal disease, when something had gone wrong, or the operation did not turn out as successful as expected. Using frightening words such as "death" and "cancer" is also considered to be part of an open and honest dialogue. The surgeons also felt responsible for "not involving patients with bad news they were not ready to receive, and feeling one's way in what it's right to inform about". They said that if the patients are taken seriously and are talked to in a way that they understand, there is seldom difficulty reaching a mutual understanding about diagnostics and treatment.

The patients are always told the truth about their disease, although it was experienced as an emotional burden for the surgeons to disclose bad news or present difficult decisions to the patients. "It's a burden to tell the patient that we will withdraw all active treatment. You feel a bit guilty; in a way you feel that it's your fault if the treatment does not succeed". They stressed that knowing the patient from previous meetings is important to them, as is having antennae for how the patients experience their life and the present situation. "If I do not manage to achieve what I am trying to do, I always tell [the patients] what the situation is. I never try to conceal anything. That will only torment them".

Talking openly with patients is also important for the surgeons in situations when they are in doubt about the right thing to do. They experienced relief if the patient had an answer to their doubts. They said that patients who are seriously ill usually have thought about life and death issues and have a conception of their condition, and that the question of withholding or withdrawing treatment seldom comes as a surprise to them. Some patients say that they have lived a good life and do not want an operation. Others strongly want an operation even though their prognoses are poor. "We often reach an agreement about ending a treatment that either does not lead to a meaningful life afterwards or leads to a life that would be experienced as a heavy burden".

#### Involvement

The surgeons said that they become personally involved with their patients, focusing on patients as persons and their quality of life as much as on their medical treatment. Being involved and knowing the patients' background and what they really want in life makes difficult ethical decisions easier to handle. The surgeons explained that caring for the patient can be felt as a personal, emotional burden. "It's not easy when people you have established a relationship with die. The only way to run away from it is to relinquish your responsibility. But that means disassociating yourself from or rejecting the patient. So you have to care, to be involved and to be a human being".

The surgeons are involved in many patients' lives and destinies and said that keeping a certain distance protects their feelings and is a way of caring for themselves. They said that keeping a certain distance is necessary in order to give the patient neutral advice and the most suitable medical treatment. The surgeons feel a responsibility to care in situations where they find it difficult, for instance when they dislike the patients' personality, behaviour, or values. They said they work hard to get involved and care for demanding and non-compliant patients.

### Social confirmation

#### Professional recognition

Ethical challenges are discussed in both formal and informal social arenas among the surgeons. All new patients are presented at the daily morning meeting between all the surgeons, including what had been done to them and why. Only questions and short objections to the patients' diagnostics and treatments are raised at these meetings or shortly after. The surgeons arrange separate meetings to discuss problematic cases. "We assemble the nurses, the anaesthesiologists, the surgeons, and even other specialists like the nephrologists when we have patients who reside a long time in the intensive care unit. In a way we create a meeting-place for the case and discuss whether we should withdraw a life-sustaining treatment or start additional treatment for a kidney failure for instance." The informal running dialogues during the day were equally important for the surgeons. "You have to make the decision yourself, but we always discuss the problem together before difficult decisions are made. The discussions do help and are experienced as mutual support". The surgeons expressed confidence in the consensus that usually is achieved in these discussions. "I know that I would be content to receive the treatment we agree upon myself".

The surgeons emphasized the importance of having a caring relationship with their colleagues. They said that talking together and giving and receiving collegial support is necessary in order to live with the personal responsibility of being in ethically difficult situations and with the emotional burden of decision making. "People say that surgeons are a bit tough and do not talk about feelings, and that may be true. But in my experience we really care for each other. Perhaps we do not go all mushy about our feelings, but we understand when someone is in difficulty. I have experienced receiving good support in such situations. Colleagues contact you and say: "Ok, listen, a couple of years back the same thing happened to me", or: "You must not take this too hard, it could have happened to anybody". That helps".

Personal and emotional support is informally and silently shared among trusted colleagues and great value is attached to it. After having presented a difficult decision about withholding treatment at a morning meeting, one of the surgeons commented: "That same afternoon, four or five of the most experienced surgeons came to me independently, and told me that they thought that what I had done was great. They said that most surgeons were not able to do what I had done. I remember it well because I think it was so well said".

#### Open dialogue

The surgeons emphasized the importance of "playing with an open hand" and that openness and honesty in the relationships with colleagues presupposes a trusting atmosphere that allows everybody to feel free to voice their opinion and be listened to. They feel that it is important that everyone who is involved in the treatment and care of the patient should have an opportunity to express their opinion and to be heard before any final decision is made. Openness and honesty are particularly important when medical errors occur or when something has gone wrong during an operation. Talking about medical errors or mistakes is considered an opportunity for learning and for improving surgical routines. "You have to have an including and accepting environment that allows you to say that you could have chosen a different solution. If there is no room for you saying something like that, then there will be a tendency to conceal it. We all make mistakes and we all make wrong deliberations and sometimes choose bad solutions. We have to live with that. Therefore it's important that we try to learn from the cases where something [erratic] happens".

The surgeons focused on the necessity of dialogue and cooperation with their colleagues. "We are used to working close together in a team and it makes these difficult situations easier to handle". They found it satisfactory to work in a hospital because "there is always someone you can ask for advice when in doubt". They said they find it easier to talk to patients about difficult treatment options when the question has been discussed with experienced colleagues or senior surgeons first. This is especially important for less experienced physicians. The surgeons also make a contrast between the importance of cooperation with colleagues and the burden of being alone and being responsible decision makers.

### Self- confirmation

#### Responsibility

The surgeons said that they experience ethically difficult situations as an important part of their everyday activities that cannot be separated from the rest. "These situations are a part of our profession that are not necessarily experienced as difficult, but are sometimes unpleasant to be in". They said they have to experience these situations personally and be involved in order to understand them and to learn to live with them.

Some ethically difficult situations are experienced as dramatic and tragic, especially when the surgeons feel the personal responsibility for saving the lives of trauma patients after major accidents. "If you are not able to cope with being in this situation and be responsible, and be the leader of the trauma team trying to save peoples' lives after a serious accident, if you cannot do that but go on wondering if you have done something wrong, then I think you will find something else to do".

The surgeons told about situations where they are alone on duty and responsible for rapid deliberations and decisions in acute and emergency situations as especially challenging. "There are no other times in this job when you feel as lonely as you do in those situations". The decision whether to continue or withdraw treatment from traumatized young patients and children is experienced as especially challenging to make alone. "It's not easy [being alone]. It's the kind of decisions you often ponder about for several days afterwards, also at home. It's the sort of decisions you really try to closely think through, and it often troubles you even after you have made a decision".

The surgeons said they have to learn to live with the unpredictable consequences of their decisions. "The practice of surgery is very specific and you feel more responsible in a way than in other areas of medicine. You have the feeling of being the direct cause when things go wrong, e.g. that you operated at the wrong time, you operated incorrectly or that you should have found the right diagnosis earlier".

#### Uncertainty

The surgeons spoke about "living with" the inherent uncertainty of surgery and emphasized that they can never be completely sure of the right thing to do in ethically difficult situations. They have to live and work with the uncertainty of the course of the disease, the patients' chances of survival, the risk of serious and fatal complications and the uncertainty of the patients' quality of life after extensive operations. The surgeons said they have to learn to live with the uncertainty of their deliberations and operations as there can be no right answer to ethical challenges, and no criteria to guide them when they make their decisions.

Living with uncertainty is experienced as both frightening and satisfying. The surgeons said that not knowing what to do in an uncertain situation, finding a way, and succeeding in their attempt to restore health or save life is a satisfying aspect of their work. They also commented that they have to live with their doubts and fears of being too active and the risk of knowingly imposing severe complications and a poor quality of life on their patients.

#### Fallibility

The surgeons said that their aim is to make all patients better. Accepting the limitation of surgery and not being able to cure a particular patient or alleviate his or her suffering is not an easy task for surgeons. They commented that it feels difficult not to be able to or not to be allowed to help patients. Sometimes they feel guilty if the treatment does not succeed, but said that they have to accept the possibility of making mistakes as an essential part of the profession. "You have to face the reality of how things are. What we are dealing with are human beings in marginal situations where things can go wrong. Everybody who is dealing with these things makes errors of deliberation and judgment. It's a part of the game that you have to live with when the margins are so tight. It would not be better if you quit. Your dearly purchased experience will be of use to nobody then".

#### Confidence

The surgeons said that they are focused on healing and curing and that they try to operate on the patients in most situations. "I believe that surgeons feel that it's good to do something, curing, and saving lives. That is what we have learned to do. We are in a profession that can do many useful things and that is the gratifying part of practicing surgery, – that you can identify a problem and do something about it".

The clinical experience of deliberating and choosing and finding workable solutions in clinical and ethical difficult situations makes the surgeons confident. "Most surgeons are action-oriented. If not, it's almost impossible to practice surgery, because you have to makes decisions all the time and be accountable for your decisions". Receiving social confirmation from patients, relatives, and colleagues when they succeed contributed to the surgeons' experience of confidence in their own decisions and actions. The surgeons also said that having the courage of your convictions and a set of personal ethical values is equally important in order to do a "proper and conscientious job". This means acting according to the patients' best interests and the standards of their profession. Their conscience helps them to decide which action is morally wrong, to make controversial decisions, and to voice personal, professional, and moral opinions to colleagues and patients.

#### Humility

The surgeons emphasized that although they deal with existential issues of life and death, they do not rule over these human conditions. "The essence is that we are not almighty. We do not save lives, we just postpone death. The only thing that is certain is that we are all going to die. You are just doing repairs and trying to help the patients to live a bit longer. " "Fortunately it's not we who rule over life and death. What we can do means less than you think and that is how it should be. I think it's important that we all acknowledge this and everyone else too".

All the surgeons said they are against active euthanasia. They also commented that a humble attitude in their work helps them acknowledge their personal and professional limitations and to recognize what they cannot and ought not do. "There is a dimension in surgery of being right about having a good reason to operate and being considerate about the benefits of carrying out an operation".

## Discussion

The surgeons in this study reasoned in a relational ethics perspective, focusing on dialogue, openness, and involvement in their relationships with patients and colleagues. While the surgeons in the interviews described their experience of being in ethically difficult situations, it seems at the same time that identity was central to their experience of ethics and their enactment of moral agency. The surgeons identified themselves by telling about their ethical experience, expressing the way they are living their identity. By narrating our lived experience, we give meaning to our experiences. We understand ourselves through the stories we tell and live, as well as those told about us and by interpreting them. Personal identity is said to be constructed through the stories we tell about our lives, stories which are in turn shaped by more general institutional, cultural, or national meta-narratives that live within the culture, of surgery, medicine, and society [40-42]. Thus, it seems that the surgeons' identity has a narrative structure and is narratively derived.

Being open and honest about all aspects of patients' treatment and care is experienced as important for the surgeons. This is even true in situations when they have to disclose bad news and tell the patient that they are not able to or did not succeed in their efforts to restore health. Openness of speech is one of the spontaneous expressions of life, designated as "utterances of life" according to the writings of Løgstrup [43]. That openness and honesty are spontaneous expressions means that they are performed in an unconstrained manner and without ulterior motives.

Human life means expressing oneself with the expectation of being met by others according to Løgstrup [43]. Openness and honesty are required in trusting relationships. When openness and honesty of speech are missing, closeness and dishonesty in the relationships result [43]. Sincerity in our relationships with others is a source of satisfaction and means being open and involved and allowing oneself to be moved and impressed by others according to Pahuus [44]. It is also probable that the surgeons' social confirmation increases when the patients meet surgeons who through dialogue turn out to be caring human beings.

Although disclosing bad news to the patient is experienced as an emotional challenge by the surgeons, concealing the truth does not seem to be an option they consider. Concealing the truth from the patient is experienced as difficult. It seems that surgeons need to share their experiences by speaking openly to patients so they can live with their demanding professional life in a satisfactory way. Therefore, honesty and openness are important because the relationships with patients are important for the surgeons' lives.

The surgeons said that it is important not to hide their uncertainty and doubts from themselves or from their patients. They accepted that uncertainty is an inherent part of their profession and realized that they often have to decide in spite of lack of scientific knowledge. They also deal with problems that may have no desirable solutions. Previous studies of surgeons [21], female physicians working in pediatrics, [30] and geriatrics [31] revealed that they are aware of and accept their own uncertainty and fallibility as inevitable in their professional lives.

The surgeons said that they sometimes feel guilty when they have to disclose the bad news to patients that the treatment did not succeed. We feel guilty when we fail to do what is required of us in a situation according to Løgstrup [43], either by not answering the ethical demand of the other or by betraying something valuable in our own life – values and beliefs we hold to be good and right. The surgeons are often in situations in which they have to use their clinical competence and practical wisdom to choose between conflicting values and obligations and being responsible for their decisions and actions. They can never know for certain in a given situation if their actions are absolutely right or wrong. They have to take risks, knowing that they may not succeed in their efforts to give patients a chance of survival.

Feeling guilty is our fundamental ethical condition as human beings according to Pahuus [44] because we have only limited possibilities at hand in particular situations and in life as a whole. Choosing one solution in a situation means excluding a multiple of other desirable possibilities. Thus, feeling guilty is a side effect of trying to give meaning and direction to our lives. Reflecting on our feelings of guilt means having a dialogue with and negotiating with our inner self, thereby evaluating what we hold to be important in our life with and for others. Feeling and being guilty is a heavy burden because human existence also contains that which is final, irreparable and cannot be changed [44]. Feeling guilty can also be understood in relation to what Ricoeur [40] has called an ethics of memory – that people never will or can and must not forget the bad or good things in history. The surgeons cannot and must not forget situations in which they failed to do the right or good thing.

The surgeons said that they experience a relief when the patients give answers to their own uncertainty and doubts about the right or good thing to do. It seems as if the surgeons are relieved when patients want to decide for themselves and take responsibility for their lives, appreciating that they do not hand over this responsibility to the surgeons. The surgeons in this study do not fit into the traditional and stereotypical picture of the paternalistic and authoritarian physicians [45]. On the contrary, the surgeons in this study seem to value being in a dialogue with patients and acknowledging their autonomy. Having to take responsibility for patients' lives is experienced as an ethical challenge and a personal burden in situations when the patients are unable to express their autonomy.

The surgeons emphasized the importance of being personally involved with the patients by focusing on patients as persons and their quality of life as much as their medical treatment. However, they experienced the challenge of finding the balance between involvement and distance in the relationships, between caring for the patients and themselves, and in giving professional and neutral information. Distance based on knowledge, skills, and experience with previous cases and situations is necessary in order to help the patients in a professional manner [43,46]. Personal involvement in a situation is necessary in order to know which fundamental values are threatened in both the patients' and the surgeons' lives. The aim of a dialogue is to abolish the distance between patients and surgeons in order to establish a space for mutual understanding. Involvement does not only mean acting according to the patients' expectations because surgeons are also responsible for their professional conduct as well as their moral integrity. Professional distance means taking the other's perspective in the situation, reflecting, deliberating, and using one's experience of similar cases. The surgeons also learned from their experiences through discussions and dialogue with experienced and trusted colleagues. They may throw new light on situations and increase the surgeons' abilities to deliberate and decide in their patients' best interests [46]. Finding the 'right' balance between involvement and distance in a situation is an expression of what Aristotle [47] calls practical wisdom (phronesis) or the practical knowledge of virtuous persons. A virtuous person is able to find the middle path between two extremes. The right and good thing to do in a particular situation involves "to the right person, to the right extent, at the right time, with the right motive, and in the right way" according to Aristotle [47].

In their narratives, the surgeons focused on cooperation and the relationships with their colleagues. They experienced being listened to and together they seem to create and recreate a collegial environment where they can express their doubts and admit their errors of deliberation and decisions.

The surgeons said that they experience taking difficult decisions alone as a burden. Working in a hospital is experienced as satisfying because they appreciate working together. They seemed to accept that they are mutually dependent on each other and appreciated the support they receive in dialogue with colleagues. Interdependence and exchange is a fundamental way of human lives according to the ethics of Løgstrup [43]. He calls this "display of life" which means that human beings are fundamentally dependent on one another and that we always are giving and receiving something in our relationships with others. This also means that each individual is challenged to take care of that part of the other's life that is in one's own power.

To be seen and listened to by colleagues, be taken seriously, and comforted when something goes wrong is an answer to fundamental human needs. Openness about personal doubts and failures also make the surgeons vulnerable. The interdependency among surgeons in this study is not experienced as a burden, but as mutual support. The surgeons' acceptance and appreciation of their collegial dependency may be an answer to why surgeons are able to stand in ethical difficult situations and live with them in a satisfying way. The dialogue with colleagues reduces the surgeons' doubts, although their doubts cannot and do not disappear.

The surgeons receive social confirmation for being good surgeons through dialogues with patients, relatives, and colleagues. Their experience of being of use to society as a whole also confirms their identity as good surgeons. To be confirmed is to be seen, listened to, and accepted by other persons. A person's identity is dependent on other persons in good or bad relationships according to Ricoeur [40], as well as an inner voice from the person's own consciousness.

The surgeons in this study emphasized the importance of dialogue with patients and colleagues. All identities are socially bestowed, socially sustained, and socially transformed and people make meanings of their actions by participating in communicative contexts. We all need recognition and social confirmation from others in order to construct our identities [41,48]. Thus the self-image of a surgeon can be maintained only in social contexts in which others are willing to recognize him or her in this identity.

There is a close relationship between identity management and values in a society since human beings usually want to be something they find valuable. What constitutes morality is created, recreated and communicated in social relations [41,49]. Narratives are a display of moral identity according to Jordens and Little [42], where the speakers present themselves as experienced, knowledgeable, and ethical. Institutional settings provide the narrative auspices under which identities are articulated. Telling stories is necessary for moral agency as we must be able to account for our actions and the actions of relevant others. We also tell stories as a sort of self-examination by self-exposure. We often find out what we think and who we are by listening to what we say.

The modern self is described as multi-voiced and dialogical and moral action is determined through a process of inner dialogue. To open up to the different voices within and enter into a dialogue, one can either reach consensus with oneself or experience conflict and dissatisfaction with oneself [41,49]. The surgeons' identities are constructed through negotiation with themselves, their patients, and colleagues within a context of social organization. The view of ourselves is shaped by the values of the larger society and what collectively is tacitly deemed to be right and good actions. Conversely, one's conception of how ethical the collective group is arises through one's personal view of what constitutes right and good actions [21,49]. Surgeons attempt to reconcile their relational experiences and their actions with their identity as moral agents as they live and practice relationally with others within the institutional values of their workplace.

The modernization and secularization of western societies emphasize the ideology of individual autonomy and freedom of choice. At the same time, the way in which social structures control and restrict individual performance and freedom becomes less visible. It is not in every person's or profession's power to choose, in other words, act as his/her conscience directs [41]. The surgeons in this study seem to have a wide range of freedom to act in a way that is confirmed both from their patients, colleagues, society and by their inner selves.

According to the surgeons, their choices of actions and ways of being in relationships with patients and colleagues have to be accepted and confirmed by their inner moral selves. They speak about "having the courage of your own conviction", "taking a stand", "having a personal set of ethical values" as important in order to "live with" the ethical challenges of surgery. The surgeons in this study revealed their identities through their narratives about being in ethically difficult situations.

Being a surgeon means having the necessary specialized knowledge, skills, and experience to perform complicated, extensive, and technically advanced operations in principally uncertain and unpredictable situations. This identity is socially sustained through dialogues with patients, relatives, and colleagues. To have an identity as a surgeon is also to understand that one is vulnerable and exposed in these relationships and to know what is at stake in one's own life as well as in the lives of the patients and colleagues.

In this study uncertainty, fallibility, and humility seem to be equally important in the surgeons' identities as are their responsibility and confidence. The social importance and benefit of their work to patients and society is confirmed both socially and by their moral selves. This seems to outweigh their personal uncertainty and vulnerability. Thus, surgeons seem to appreciate their work and have learned to live with, and to a certain extent even appreciate, its most difficult dimensions. The meaning of being a surgeon is to be able to live with the tensions of contradictions, ambivalence, dilemmas, and paradoxes in their practice and to find workable solutions in these situations.

## Competing interests

The author(s) declare that they have no competing interests.

## Authors' contributions

KT participated in the design of the study, carried out the interviews, participated in the analysis and completed the manuscript.

AN read the interviews, participated in the analysis and helped to draft the manuscript.

VS participated in the design of the study, read the interviews, participated in the analysis and helped to draft the manuscript.

All authors have read and approved the final manuscript.

## Pre-publication history

The pre-publication history for this paper can be accessed here:


